# The Secretome of Human Deciduous Tooth-Derived Mesenchymal Stem Cells Enhances In Vitro Wound Healing and Modulates Inflammation

**DOI:** 10.3390/pharmaceutics17080961

**Published:** 2025-07-25

**Authors:** Thais Simião Payão, Vanessa Pellegrini, Joseane Morari, Gisele Mara Silva Gonçalves, Maria Carolina Ximenes de Godoy, Alessandra Gambero, Leonardo O. Reis, Lício Augusto Velloso, Eliana Pereira Araújo, Lívia Bitencourt Pascoal

**Affiliations:** 1 School of Life Sciences, Faculty of Veterinary Medicine, Pontifical Catholic University of Campinas (PUC Campinas), São Paulo 13060-904, Brazil; thais.sp3@puccampinas.edu.br (T.S.P.); vpvanpellegrini@gmail.com (V.P.); 2 ImmunOncology, Pontifical Catholic University of Campinas, São Paulo 13060-904, Brazil; alegambero@gmail.com (A.G.); reisleo@unicamp.br (L.O.R.); 3 UroGen, National Institute of Science, Technology and Innovation in Genitourinary Cancer (INCT), Campinas, São Paulo 13060-904, Brazil; 4 Laboratory of Cell Signaling, Obesity and Comorbidities Research Center, School of Medical Sciences, University of Campinas, São Paulo 13083-864, Brazil; morarij@gmail.com (J.M.); lavellos@unicamp.br (L.A.V.); paeliana@gmail.com (E.P.A.); 5 Postgraduate Program in Health Sciences, School of Life Sciences, Pontifical Catholic University of Campinas (PUC Campinas), São Paulo 13060-904, Brazil; gmsg@puc-campinas.edu.br (G.M.S.G.); maria.cxg@puccampinas.edu.br (M.C.X.d.G.); 6 UroScience, University of Campinas, Campinas, São Paulo 13083-887, Brazil; 7 Faculty of Nursing, University of Campinas, São Paulo 13083-887, Brazil

**Keywords:** human dental pulp-derived mesenchymal stem cells, secretome, wound healing, skin regeneration, anti-inflammatory effect

## Abstract

**Background/Objectives:** Chronic wounds represent a significant clinical and public health challenge due to impaired tissue repair and high associated morbidity. This study investigates the therapeutic potential of the secretome derived from human mesenchymal stem cells obtained from the pulp of deciduous teeth (hDP-MSCs) in promoting skin wound healing. **Methods:** After confirming the mesenchymal identity and multipotent differentiation potential of hDP-MSCs by using flow cytometry and histological staining, the effects of the secretome on human keratinocyte (HaCaT) cultures were evaluated. **Results:** Scratch assays, performed under high- and low-glucose conditions, demonstrated that the secretome significantly promoted keratinocyte migration and wound closure without compromising cell viability. Additionally, the secretome modulated the expression of key genes involved in inflammation and tissue regeneration, including IL-1β, TNF-α, TGF-β1, and VEGF-α, in a time-dependent manner. Under inflammatory conditions induced by lipopolysaccharide, co-treatment with the secretome significantly reduced TNF-α expression and increased TGF-β1 expression, suggesting an anti-inflammatory effect. **Conclusions:** These findings indicate the potential of the hDP-MSC-derived secretome as a promising cell-free therapeutic strategy capable of accelerating skin regeneration and modulating the inflammatory response during the wound healing process.

## 1. Introduction

Wound healing is a complex and dynamic process that involves the coordinated activation of cellular, molecular, and biochemical mechanisms aimed at restoring tissue integrity and homeostasis. This process depends on the interaction between keratinocytes, fibroblasts, endothelial cells, inflammatory mediators, and components of the extracellular matrix [1,2]. However, in unfavorable clinical situations, such as pressure ulcers, extensive burns, diabetes mellitus, or peripheral vascular diseases, this process can be interrupted or significantly delayed, resulting in poor or chronic healing [3,4,5].

Inflammation plays a dual role in this process, as it is essential for initiating the wound healing cascade by promoting immune cell recruitment, microbial clearance, and tissue debridement; however, when excessive or prolonged, it can lead to tissue damage, fibrosis, and chronic wound development. Therefore, regulating the inflammatory response is a crucial therapeutic target to achieve balanced healing and avoid pathological outcomes [6,7].

Chronic wounds, resulting from this imbalance in healing, represent a significant clinical challenge and a global public health issue. It is estimated that in the United States alone, approximately 2.5 million cases of pressure ulcers are treated annually, with an estimated cost of up to USD 17.8 billion. In addition to the economic impact, these lesions are associated with high morbidity and impaired quality of life, especially in vulnerable populations such as the elderly, hospitalized individuals, immobilized patients, those who are malnourished, or those with compromised tissue perfusion [8,9]. In light of this scenario, it becomes essential to identify new therapeutic strategies that promote effective skin regeneration, focusing on the functional and morphological restoration of the damaged tissue.

In this context, mesenchymal stem cell (MSC) therapies have emerged as a promising alternative in regenerative medicine. MSCs are multipotent cells with remarkable self-renewal capacity, differentiation into multiple cell lineages, and immunomodulatory properties that promote the regulation of the inflammatory response [10,11,12]. They can be isolated from various tissue sources, including bone marrow, adipose tissue, the placenta, the umbilical cord, and deciduous dental pulp. Among these sources, human dental pulp-derived mesenchymal stem cells (hDP-MSCs) stand out due to their non-invasive collection process, high in vitro proliferation rate, and significant secretory activity, characteristics that make them particularly attractive for regenerative medicine applications [12,13,14,15].

More recently, the scientific community has focused on studying a new biological regulation methodology involving communication between cells through their secreted substances, known as the secretome. The secretome is considered a cell-free therapy approach, defined as a set of bioactive factors secreted by cells into the extracellular space, including cytokines, growth factors, enzymes, lipids, nucleic acids, and extracellular vesicles. These elements act in an integrated manner to modulate inflammation, stimulate angiogenesis, promote cell proliferation, and enhance tissue regeneration. In vitro and in vivo studies have demonstrated that the secretome of MSCs exerts beneficial effects on skin repair, autoimmune diseases, degenerative processes, and tissue aging [16,17,18,19,20,21].

Given the need for effective and safe therapeutic alternatives for the treatment of chronic skin wounds, the present study aimed to evaluate the therapeutic potential of the secretome derived from human mesenchymal stem cells obtained from deciduous dental pulp in the in vitro skin healing process. For this purpose, assays were conducted with human keratinocyte (HaCaT) cultures to investigate the effects of the secretome on cell migration and the modulation of the inflammatory response, with and without lipopolysaccharide (LPS) stimulation. This study aims to contribute to the advancement of knowledge in the field of regenerative medicine and provide insights into the development of innovative therapies based on biological products derived from stem cells.

## 2. Materials and Methods

**Figure 1:**
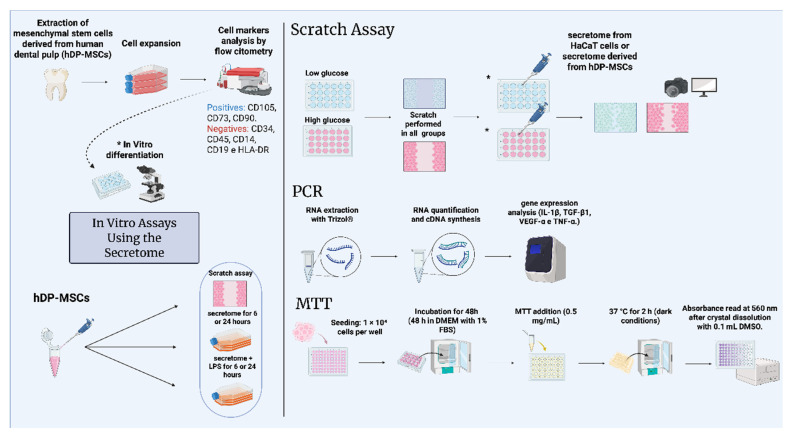
Schematic representation of the in vitro treatment of keratinocyte cells with the secretome derived from human mesenchymal stem cells. The figure illustrates the isolation of mesenchymal stem cells, phenotypic characterization, differentiation methodology, and cell culture for subsequent secretome collection. The secretome was applied in scratch assays, treatments with secretome and/or LPS, and cell viability analysis. Scratch assay, real-time PCR, and MTT assays were performed. * In vitro differentiation. The diagram was created using BioRender.com. Created in BioRender. Payão, T. (2025) https://BioRender.com/e4ub5w8.

All experimental steps and methodological procedures described in this study are schematically illustrated in Figure 1, providing an overview of the complete experimental workflow.

### 2.1. Sampling and Isolation of Mesenchymal Stem Cells Derived from Deciduous Dental Pulp

Human dental pulp tissues were collected from seven pediatric patients undergoing elective tooth extraction at the Pediatric Dentistry Clinic of the Pontifical Catholic University of Campinas (PUC-Campinas), following written informed consent obtained from their legal guardians. The study was approved by the Research Ethics Committee of PUC-Campinas under protocol number CAAE: 64109822.0.0000.5481.

Immediately after extraction, the tissues were stored in phosphate-buffered saline (PBS) supplemented with Antibiotic-Antimycotic solution (Gibco, Thermo Fisher Scientific, Waltham, MA, USA; cat. no. 15240096) at 4 °C for up to 24 h. For cell isolation, the samples were finely minced using sterile scissors or a scalpel and enzymatically digested with type II collagenase (Sigma-Aldrich, St. Louis, MO, USA) for 40 min at 37 °C under continuous agitation (250 rpm using a Microplate Shaker KJ-201C; Global Trade Technology, São Paulo, Brazil). Following digestion, the cell suspensions were centrifuged, and the resulting pellets were resuspended and plated in low-glucose Dulbecco’s Modified Eagle Medium (DMEM-LG; Gibco, Thermo Fisher Scientific, Waltham, MA, USA) supplemented with 20% fetal bovine serum (FBS; Gibco, Thermo Fisher Scientific, Waltham, MA, USA) and 1% Antibiotic-Antimycotic solution (Gibco, Thermo Fisher Scientific, Waltham, MA, USA cat. no. 15240096) using a modified protocol based on [22]. Cultures were maintained at 37 °C in a humidified atmosphere containing 5% CO_2_. Cells were expanded up to passage three and cryopreserved at −80 °C for subsequent analyses.

#### 2.1.1. Phenotyping of Human Mesenchymal Stem Cells Isolated from Deciduous Dental Pulp

The phenotypic characterization of hDP-MSCs was performed in accordance with current guidelines and the criteria established in the recent literature [23,24]. The MSC lines were assessed for specific surface markers via flow cytometry, evaluating the expression of CD73, CD90, CD105, CD45, CD34, and HLA-DR. Monoclonal antibodies were obtained from BD Biosciences and used as follows: anti-CD73 (cat. no. 550257), anti-CD90 (cat. no. 581969), anti-CD105 (cat. no. 560839), anti-CD45 (cat. no. 555493), anti-CD34 (ref. no. 348053), and anti-HLA-DR (cat. no. 555560).

Flow cytometry analysis was performed using a Becton Dickinson FACSCalibur flow cytometer (BD Biosciences, Oxford, UK) equipped with a 488 nm argon excitation laser (15 mW). A total of 10,000 events were acquired per sample. Immune cell populations were excluded based on forward and side scatter profiles. Data were analyzed for mean fluorescence intensity to assess marker expression.

#### 2.1.2. Confirmation of Human Mesenchymal Stem Cell Differentiation Potential

Following flow cytometry characterization, hDP-MSCs were seeded in 12-well plates (Corning^®^, Corning Inc., Corning, NY, USA) and maintained at 37 °C in a humidified atmosphere with 5% CO_2_ until they reached approximately 90% confluence. To assess their multipotent differentiation potential, the cells were subjected to adipogenic, osteogenic, and chondrogenic induction using the StemPro^®^ differentiation kits (Gibco™, Thermo Fisher Scientific, Waltham, MA, USA), following the manufacturer’s instructions. Differentiation was evaluated at days 2, 7, and 14 following the initiation of culture in specific induction media.

For adipogenic differentiation, cells were fixed in 4% formaldehyde for 30 min, rinsed with PBS, and stained with Oil Red O solution for 30 min to identify intracellular lipid droplets. For osteogenic differentiation, cells were fixed in 4% formaldehyde for 30 min and were subsequently stained with 2% Alizarin Red S (pH 4.1–4.3) for 3 min to detect calcium-rich extracellular matrix deposits. Chondrogenic differentiation was assessed by fixing the cells in 4% formaldehyde, followed by staining with 1% Alcian Blue in 0.1 N HCl for 30 min. Excess stain was removed with three washes of 0.1 N HCl, and cells were then rinsed with distilled water to neutralize the pH.

Representative images of differentiated cells were captured using a Zeiss^®^ Axioplan 2 microscope (Carl Zeiss Microscopy GmbH, Jena, Germany) equipped with an Olympus^®^ DP-72 camera (Olympus Corporation, Tokyo, Japan) and analyzed using CellSens^®^ software (Olympus Corporation, Tokyo, Japan), with 5× and 20× objective lenses.

#### 2.1.3. Cell Culture and Collection of Conditioned Medium (Secretome)

Third-passage hDP-MSCs were cultured in low-glucose Dulbecco’s Modified Eagle Medium (DMEM-LG; Gibco) supplemented with 20% fetal bovine serum (FBS) and 1% Antibiotic-Antimycotic solution (Gibco, cat. no. 15240096). Cells were maintained in 75 cm^2^ culture flasks under standard conditions (37 °C, 5% CO_2_) until reaching approximately 90% confluence. Upon reaching this stage, the culture medium was replaced with freshly prepared supplemented medium.

The conditioned medium (secretome) was collected every five days, filtered through a 0.22 μm membrane filter, aliquoted, and stored at −80 °C until further use.

#### 2.1.4. HaCaT Cell Culture, Secretome Treatment, and Inflammation Induction

HaCaT keratinocyte cells were seeded at a density of 50,000 cells per well in 24-well plates and maintained under standard culture conditions (37 °C, 5% CO_2_, humidified atmosphere) for five days without medium replacement. The conditioned medium (secretome) from HaCaT cells, used as the control treatment (CTR group), was collected every five days, filtered through a 0.22 μm membrane, aliquoted, and stored at −80 °C until use.

For experimental treatments, cells were exposed either to the secretome derived from human dental pulp mesenchymal stem cells (SeCR group) or to HaCaT-derived secretome (CTR group), both previously prepared and stored as described above. Treatments were administered by adding 80 μL of secretome to 320 μL of complete DMEM—either low-glucose or high-glucose formulations—supplemented with 10% fetal bovine serum (FBS) and 1% Antibiotic-Antimycotic solution. This yielded a final treatment volume of 400 μL per well, corresponding to a secretome concentration of 20% (*v/v*).

To induce an inflammatory response, HaCaT cells were stimulated with lipopolysaccharide (LPS; Merck, Cat. No. L2630—10 mg) at concentrations of 2, 4, and 6 μg/mL. The concentration of 4 μg/mL was selected for subsequent experiments, as it consistently induced a measurable inflammatory response without compromising cell viability.

#### 2.1.5. Scratch Assay and Quantification of Scratch Closure in HaCaT Cells

The regenerative potential of the secretome derived from human dental pulp mesenchymal stem cells (hDP-MSCs) was evaluated with an in vitro scratch assay using HaCaT keratinocyte monolayers. HaCaT cells were seeded in 24-well plates and cultured in low-glucose DMEM supplemented with 10% fetal bovine serum and 1% Antibiotic-Antimycotic solution until reaching full confluence. A linear scratch was then created in each well using a sterile pipette tip, followed by gentle washing with PBS to remove cell debris and detached cells [25].

Following scratch induction, cells were treated under two experimental conditions: the control group (CTR group) received conditioned medium from HaCaT cells, while the experimental group (SeCR group) was treated with secretome obtained from hDP-MSCs. Wound closure and cell migration were monitored at 0, 24, and 48 h post-treatment using phase-contrast microscopy with an inverted microscope.

Quantitative analysis of cell migration and wound repair was performed by measuring the scratch area at each time point using ImageJ software (version 1.49; National Institutes of Health, Bethesda, MD, USA). Wound closure was calculated using the formula Wound closure (%) = [(A_0_ − Aₜ)/A_0_] × 100, where *A*_0_ represents the wound area at time zero and *Aₜ* corresponds to the area at 24 or 48 h post-treatment [25].

All experiments were conducted in triplicate. To better replicate both physiological (normoglycemic) and pathological (hyperglycemic) conditions relevant to skin repair, particularly in the context of impaired wound healing observed in diabetic patients, the scratch assay was also conducted using high-glucose DMEM. This approach allowed us to evaluate the therapeutic potential of the hDP-MSC-derived secretome under distinct metabolic environments. The corresponding results are provided in Supplementary Figure S1.

#### 2.1.6. RT-qPCR Analysis

For transcriptional gene expression analyses, total RNA was extracted using TRIzol™ reagent (Invitrogen™, Thermo Fisher Scientific, Waltham, MA, USA), following the manufacturer’s protocol. RNA concentration and purity were assessed using a NanoDrop™ spectrophotometer (Thermo Fisher Scientific, Waltham, MA, USA), and only samples with acceptable 260/280 nm ratios were used. cDNA synthesis was performed using a High-Capacity cDNA Reverse Transcription Kit (Applied Biosystems™, (Thermo Fisher Scientific, Waltham, MA, USA) in a thermocycler, with a final cDNA concentration of 200 ng. This cDNA was diluted to the necessary concentration to amplify each transcribed gene efficiently.

#### 2.1.7. Quantitative PCR

Real-time PCR reactions were performed in 8 µL volumes containing diluted cDNA, primers, and reagents according to the manufacturer’s recommendations. Cycling conditions included 50 °C for 10 min, 95 °C for 2 min, followed by 45 cycles at 95 °C for 5 s and 60 °C for 30 s. Target genes included *TNF-α* (Hs.PT.58.45380900), *IL-1β* (Hs.PT.58.1518186), *VEGF* (Hs.PT.58.1149801), e *TGF-β1* (Hs.PT.58.39813975), using gene-specific primers obtained from IDT DNA Technologies. The human endogenous control gene *PPIA* (Hs04194521_s1; Thermo Fisher Scientific) was used for the normalization of target gene expression. Gene expression was normalized to an internal control using the ΔΔCt method, analyzing fluorescence values normalized to the endogenous controls with the 7500 System SDS Software version 1.4 (Applied Biosystems^®^, Thermo Fisher Scientific, Waltham, MA, USA) [26].

#### 2.1.8. Cell Viability Analysis Using an MTT Assay

Cell viability was assessed using an MTT assay (3-(4,5-dimethylthiazol-2-yl)-2,5-diphenyl-2H-tetrazolium bromide) on HaCaT cells. Cells were seeded in 12-well plates at a density of 1 × 10^4^ cells per well and cultured in DMEM with low or high glucose supplemented with 1% fetal bovine serum (FBS). After 48 h of incubation, culture medium was removed, and MTT (0.5 mg/mL) was added to each well. Cells were incubated at 37 °C for 2 h in the dark, after which the MTT-containing medium was discarded. Formazan crystals were dissolved with 0.1 mL DMSO per well. Absorbance was measured at 490 nm using a Varioskan Flash microplate reader (Thermo Scientific, Waltham, MA, USA) [25,27].

#### 2.1.9. Statistical Analysis of Results

Results are expressed as mean +/− standard error of the mean. Leven’s test was initially used to assess variance homogeneity. For comparisons between two groups, Student’s *t*-test for independent samples was applied. For comparisons among three or more groups, analysis of variance (ANOVA) was performed, followed by Bonferroni’s post hoc test. A significance level of *p* < 0.05 was adopted. Statistical analysis and graph generation were performed using GraphPad Prism (v8.0; GraphPad Software, CA, USA).

## 3. Results

### 3.1. Confirmation of hDP-MSC Identity as Human Mesenchymal Stem Cells

In the initial phase of the study, third-passage human dental pulp mesenchymal stem cells (hDP-MSCs) were incubated with fluorophore-conjugated antibodies specific to surface markers and were subsequently analyzed using flow cytometry. The evaluated markers included CD105, CD90, CD73, HLA-DR, CD45, and CD34.

**Figure 2:**
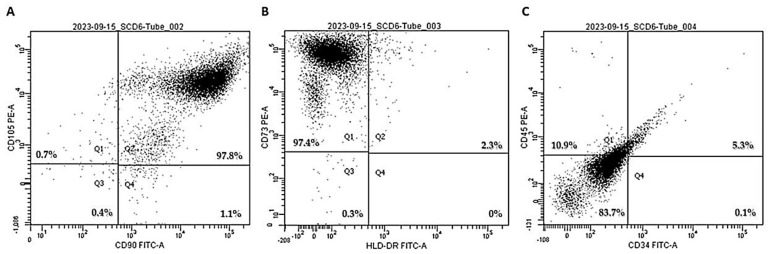
Representative image of the qualitative analysis of specific surface markers for the characterization of mesenchymal stem cells. The expression of cell surface proteins, including (**A**) CD105, CD90, (**B**) CD73, HLA-DR, (**C**) CD45, and CD34, was evaluated. The results were analyzed based on the mean fluorescence intensity (MFI) of the samples.

Flow cytometry analysis demonstrated that hDP-MSCs were highly positive for classical mesenchymal markers, including CD105 and CD90 (both 97.8%; Figure 2A), as well as CD73 (97.4%; Figure 2B). Additionally, the cells were entirely negative for HLA-DR (100%; Figure 2B). Moreover, they exhibited low expression of hematopoietic and immunological markers, with 83.7% of the cells negative for both CD45 and CD34 (Figure 2C).

These results confirm that the isolated cell population exhibits the characteristic phenotypic profile of mesenchymal stem cells, in line with the minimal criteria established by the International Society for Cellular Therapy (ISCT). The absence of hematopoietic and immune markers, combined with the strong expression of MSC-associated surface antigens, supports the successful isolation and characterization of hDP-MSCs.

**Figure 3:**
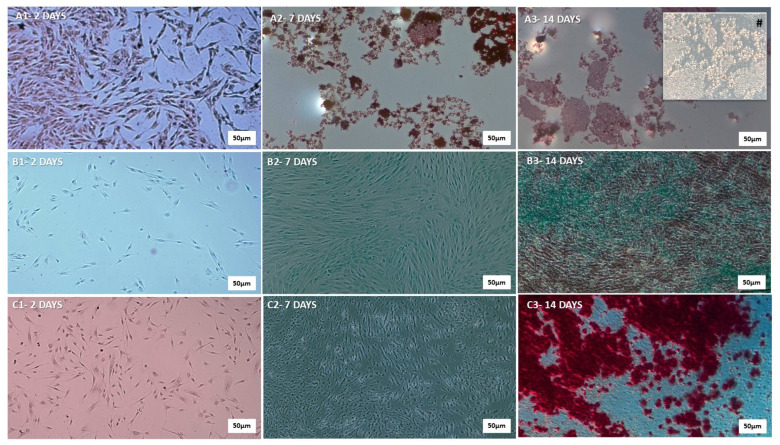
Qualitative representation of adipogenic, chondrogenic, and osteogenic differentiation of human dental pulp mesenchymal stem cells (hDP-MSCs). (**A**) Adipogenic differentiation: A1—day 2, A2—day 7, A3—day 14, illustrating the progressive accumulation of intracellular lipid droplets. (**B**) Chondrogenic differentiation: B1—day 2, B2—day 7, B3—day 14, indicating the gradual synthesis of cartilage matrix components, such as type II collagen and proteoglycans. (**C**) Osteogenic differentiation: C1—day 2, C2—day 7, C3—day 14, demonstrating progressive production of type I collagen, a key component of the bone matrix. Images were captured at higher magnifications (5× and 20×) using a Zeiss^®^ Axioplan 2 microscope equipped with an Olympus^®^ DP-72 digital camera and CellSens^®^ software. Scale Bar 500 µm. These procedures ensured accurate visualization of the differentiated cell types. # 20×.

The mesenchymal stem cells were cultured and subjected to differentiation protocols to evaluate their multipotent potential (Figure 3). The hDP-MSCs demonstrated the ability to differentiate into the three classical mesenchymal lineages: adipocytes (Figure 3A), chondrocytes (Figure 3B), and osteocytes (Figure 3C). Multipotency was confirmed using lineage-specific histological staining at 2 (1), 7 (2), and 14 days (3) post-induction. Adipogenic differentiation was evidenced by the accumulation of intracellular lipid droplets, visualized using Oil Red O staining. Chondrogenic differentiation was confirmed by the presence of sulfated proteoglycans, highlighted using Alcian Blue staining. Osteogenic differentiation was verified using calcium-rich extracellular matrix deposition and demonstrated using Alizarin Red staining. In all conditions, the cells exhibited characteristic morphology and staining patterns consistent with each specific lineage, thereby confirming the tri-lineage differentiation potential and multipotency of the hDP-MSCs.

### 3.2. Secretome Stimulate Cell Migration and Proliferation in Scratch Assay with HaCaT Keratinocytes

Following the characterization and confirmation of the multipotency of human dental pulp-derived mesenchymal stem cells (hDP-MSCs), the next phase of the study evaluated the efficacy of their secretome in promoting wound healing in HaCaT keratinocytes under different glucose conditions.

**Figure 4:**
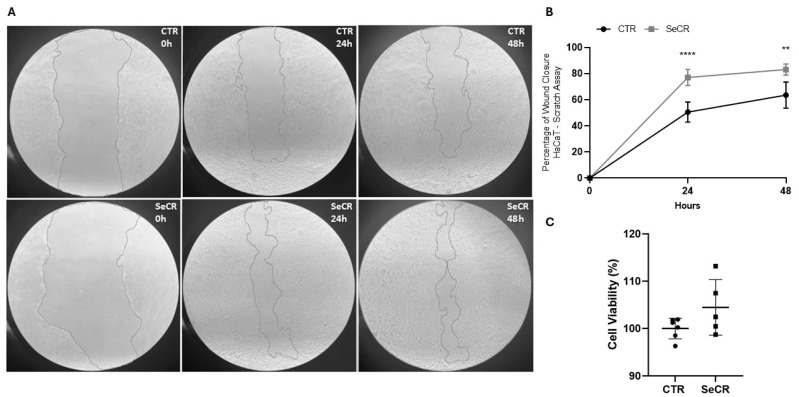
Effect of the secretome on wound closure in HaCaT cells at 0 h, 24 h, and 48 h. (**A**) Representative images of the scratch assay showing cell migration in the control group (CTR) and the secretome-treated group (SECR) at different time points. (**B**) Graph depicting the percentage of wound closure after 24 h and 48 h. (**C**) The effect of the secretome on keratinocyte viability was assessed after 24 and 48 h of treatment, as evidenced by the MTT assay. Secretome-treated cells exhibited significantly greater wound closure compared to the control group. *n* = 5. ** *p* < 0.01; **** *p* < 0.0001.

In high-glucose medium, the scratch assay demonstrated a significant enhancement in cell migration in the secretome-treated groups compared to controls (Figure 4A). HaCaT cells treated with the secretome exhibited 77.05% wound closure after 24 h, compared to 50.58% in the control group (*p* < 0.0001; Figure 4B). At 48 h, wound closure was 83.15% in the treated group, versus 63.57% in controls (*p* = 0.0013; Figure 4B). These results confirm that the hDP-MSC-derived secretome significantly enhances keratinocyte migration and accelerates in vitro wound closure.

Next, it was investigated whether treatment with the secretome could modulate the cellular viability of human keratinocyte HaCaT cells. The secretome did not affect keratinocyte viability after 24 and 48 h of treatment (*p* = 0.1170; Figure 4C). Furthermore, the secretome was not cytotoxic to human keratinocytes at any of the incubation periods.

Similarly, in low-glucose medium, the scratch assay demonstrated a significant enhancement in cell migration in the secretome-treated groups compared to controls (Supplementary Figure S1A). Wound closure after 24 h reached 79.51% in the secretome-treated group versus 53.32% in the control group (*p* < 0.0001; Supplementary Figure S1B). After 48 h, wound closure was 87.86% in the treated group, significantly higher than the 62.02% observed in the controls (*p* = 0.002; Supplementary Figure S1B), and the secretome did not alter the cellular viability of human keratinocyte HaCaT cells in low-glucose medium (*p* = 0.1028; Supplementary Figure S1C).

These findings indicate that the secretome enhances in vitro cell migration, promoting faster wound closure, a key process in tissue healing. These results support the hypothesis that the hDP-MSC-derived secretome contains bioactive factors capable of modulating cellular responses.

### 3.3. Expression Profile of Key Inflammatory and Regenerative Genes Induced by the hDP-MSC-Derived Secretome

**Figure 5:**
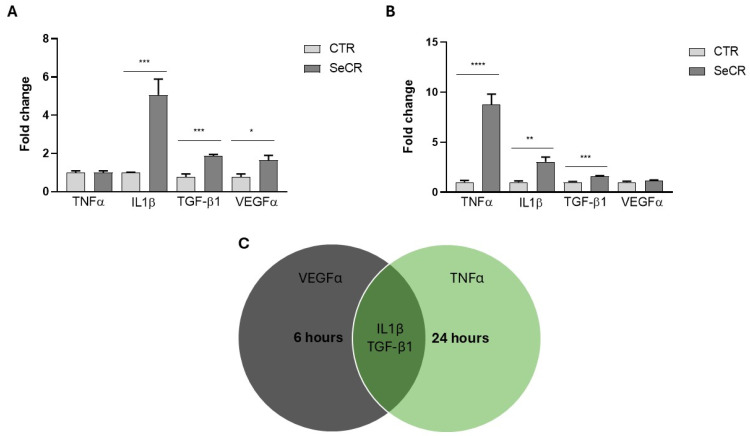
Gene expression profile of mediators related to the skin wound healing process. (**A**) Gene expression of TNF-α, IL-1β, TGF-β1, and VEGF in HaCaT cells treated or not treated with secretome for 6 h. (**B**) Gene expression of TNF-α, IL-1β, TGF-β1, and VEGF in HaCaT cells treated or not treated with secretome for 24 h. (**C**) Venn diagram illustrating the temporal modulation of genes: those with increased expression at 6 and 24 h, and those commonly upregulated at both time points. *n* = 6. * *p* < 0.05; ** *p* < 0.01; *** *p* < 0.001; **** *p* < 0.0001.

Subsequently, the gene expression profile of classical mediators involved in the wound healing process was evaluated after 6 and 24 h of cell culture with the MSC-derived secretome. After 6 h, significant upregulation of interleukin-1 beta (IL-1β) (*p* < 0.001), transforming growth factor beta 1 (TGF-β1) (*p* < 0.001), and vascular endothelial growth factor (VEGFα) (*p* < 0.05) was observed in the secretome-treated group compared to the control (Figure 5A). Treatment with the secretome for 6 h did not modulate TNF-α gene expression compared to the control group (*p* > 0.99; Figure 5A).

At 24 h, significant modulation of tumor necrosis factor alpha (TNF-α) (*p* < 0.0001), IL-1β (*p* = 0.01), and TGF-β1 (*p* < 0.001) was also detected in the treated group relative to controls (Figure 5B). Treatment with the secretome for 24 h did not modulate VEGFα gene expression compared to the control group (*p* = 0.3067; Figure 5B). These results demonstrate that the secretome exerts a time-dependent regulatory effect on the expression of key mediators of inflammation and tissue regeneration, as shown in the representative Figure 5C, reinforcing its potential role in modulating the wound healing process.

### 3.4. Effect of the Secretome on the Modulation of the Inflammatory Response in LPS-Stimulated Keratinocytes

An experiment was conducted using HaCaT keratinocyte cells subjected to an inflammatory stimulus with lipopolysaccharide (LPS) at a concentration of 4 µg/mL for 6 h, in association with treatment using the hDP-MSC-derived secretome, to evaluate its potential modulatory effect on cellular inflammatory response.

**Figure 6:**
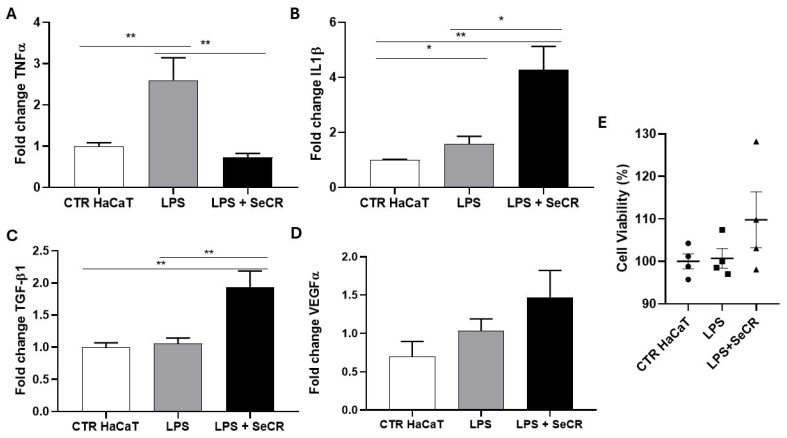
Gene expression profile of mediators related to the skin wound healing process following inflammatory stimulation with LPS after 6 h. Evaluation of the expression of TNF-α (**A**), IL-1β (**B**), TGF-β1 (**C**), and VEGF-α (**D**) in HaCaT cells from the control group (CTR), LPS-treated group (LPS), and the group treated with LPS followed by secretome (LPS + SeCR) after 6 h of incubation. (**E**) The effect of the LPS and LPS + secretome on keratinocyte viability was assessed after 6 h of treatment, as evidenced by the MTT assay. *n* = 6. * *p* < 0.05; ** *p* < 0.01.

Gene expression analysis of inflammatory cytokines revealed that treatment with LPS alone (LPS group) led to a significant upregulation of TNF-α expression compared to the untreated control group (CTR HaCaT), confirming activation of the inflammatory response (*p* = 0.0084; Figure 6A). However, co-treatment with the secretome (LPS + SeCR group) resulted in a marked reduction in TNF-α levels, approaching those observed in the control group, suggesting the potential anti-inflammatory effect of the secretome (*p* = 0.0099; Figure 6A).

For IL-1β, a significant increase in expression was observed in both the LPS-treated and LPS + SeCR groups compared to the CTR HaCaT (*p* = 0.044 and *p* = 0.0011, respectively; Figure 6B). Furthermore, it was observed that the LPS + SeCR group exhibited increased IL-1β levels compared to the LPS group (*p* = 0.0105; Figure 6B). These findings, combined with the observation that the secretome alone modulated IL-1β expression after 6 h of treatment without LPS (Figure 5A), suggest that IL-1β may act not only as a classical pro-inflammatory mediator, but also as a key regulator during the phases of wound healing. Therefore, the secretome may play a more complex regulatory role in IL-1β expression.

Regarding TGF-β1 expression (Figure 6C), no significant difference was observed between the CTR HaCaT and LPS groups (*p* = 0.6224). However, a significant increase in gene expression was detected after 6 h of treatment with LPS + secretome compared to the CTR HaCaT group (*p* = 0.0044). Additionally, TGF-β1 expression was also significantly higher in the LPS + secretome group compared to the LPS-only group (*p* = 0.0073). This elevated expression remained stable even in the presence of LPS, indicating that TGF-β1 induction by the secretome is not significantly affected by the inflammatory challenge under the tested conditions.

Finally, VEGF-α expression levels did not show significant changes in any of the experimental groups (CTR HaCaT vs. LPS: *p* = 0.2105; CTR HaCaT vs. LPS + secretome: *p* = 0.0828; LPS vs. LPS + secretome: *p* = 0.2793; Figure 6D). These results suggest a low responsiveness of this angiogenic factor to the inflammatory stimulus under the conditions tested.

Importantly, cell viability was evaluated using an MTT assay under the same experimental conditions. The results confirmed that the LPS concentration used (4 µg/mL) effectively triggered an inflammatory response without inducing cytotoxicity or apoptosis in HaCaT cells after 6 h (Figure 6E). Moreover, treatment with the hDP-MSC-derived secretome did not reduce cell viability, even in the presence of LPS. These findings reinforce the cytocompatibility of the secretome and its potential therapeutic safety profile in inflamed tissue environments.

### 3.5. Secretome-Mediated Modulation After 24 h LPS Exposure

Following a similar experimental approach to the 6 h assay, we evaluated the effect of the hDP-MSC-derived secretome on keratinocytes under inflammatory conditions induced by lipopolysaccharide (LPS) for 24 h.

**Figure 7:**
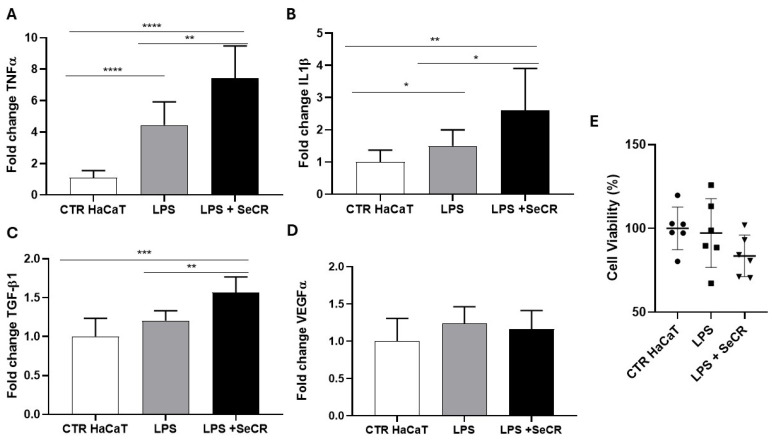
Gene expression profile of mediators related to the skin wound healing process following inflammatory stimulation with LPS after 24 h. Evaluation of the expression of TNF-α (**A**), IL-1β (**B**), TGF-β1 (**C**), and VEGF-α (**D**) in HaCaT cells from the control group (CTR), LPS-treated group (LPS), and the group treated with LPS followed by secretome (LPS + SECRET), after 24 h of incubation. (**E**) The effect of the LPS and LPS + secretome on keratinocyte viability was assessed after 24 h of treatment, as evidenced by the MTT assay. *n* = 6. * *p* < 0.05; ** *p* < 0.01; *** *p* < 0.001; **** *p* < 0.0001.

Gene expression analysis of inflammatory cytokines revealed that treatment with LPS alone (LPS group) resulted in a significant upregulation of TNF-α compared to the untreated control group (CTR HaCaT), confirming the sustained activation of the inflammatory response (*p* < 0.0001; Figure 7A). Surprisingly, co-treatment with the secretome (LPS + SeCR group) also led to significantly elevated TNF-α levels compared to both the CTR group (*p* < 0.0001) and the LPS-only group (*p* = 0.0088; Figure 7A), indicating a potential time-dependent shift in the secretome’s regulatory effect.

For IL-1β, a significant increase in gene expression was observed in the LPS-treated group compared to the control group (*p* = 0.0043), as well as in the LPS + SeCR group relative to the control group (*p* = 0.0053). Moreover, IL-1β expression was significantly higher in the LPS + SeCR group than in the LPS-only group (*p* = 0.0434; Figure 7B), suggesting a continued or enhanced activation of this cytokine following prolonged secretome exposure under inflammatory conditions.

Regarding TGF-β1 expression (Figure 7C), the LPS-only group did not differ significantly from the CTR group (*p* = 0.0628). However, cells treated with LPS + secretome showed a robust increase in TGF-β1 expression compared to the control group (*p* = 0.0002) and to the LPS-only group (*p* = 0.0016), reinforcing the notion that the secretome may actively promote regenerative pathways even during ongoing inflammation.

As for VEGF-α, no statistically significant differences were observed among the experimental groups (CTR HaCaT vs. LPS: *p* = 0.0995; CTR HaCaT vs. LPS + SeCR: *p* = 0.2590; LPS vs. LPS + SeCR: *p* = 0.5455; Figure 7D), suggesting that the angiogenic response remains relatively unaltered under the tested conditions after 24 h of LPS stimulation.

Complementarily, MTT assays performed after 24 h of exposure confirmed that the LPS concentration used did not induce loss of cell viability (Figure 7E). In addition, cells treated with the secretome maintained high viability levels under inflammatory conditions, indicating that the secretome did not exhibit cytotoxic effects over time. These results further support the safety and cytocompatibility of the hDP-MSC-derived secretome under both acute and prolonged inflammatory stress.

## 4. Discussion

Cutaneous wound healing, particularly in cases of extensive, deep, or chronic injuries, remains a major challenge in regenerative medicine. Delayed wound repair, defined as healing that extends beyond three weeks, is associated with a higher risk of complications, such as infection, persistent inflammation, impaired re-epithelialization, and excessive scar formation. These complications are further exacerbated under systemic conditions such as diabetes mellitus, where hyperglycemia impairs keratinocyte migration, angiogenesis, and extracellular matrix remodeling [2,3,4].

Mesenchymal stem cells (MSCs) have emerged as promising agents in tissue repair due to their immunomodulatory and regenerative capabilities [28,29]. In this context, secretome-based therapies are gaining traction as cell-free alternatives, offering safety, scalability, and potent bioactivity mediated by soluble factors, extracellular vesicles, and cytokines released by MSCs.

The present study confirms the successful isolation and characterization of third-passage human dental pulp-derived MSCs (hDP-MSCs), which met the minimal criteria established by the International Society for Cellular Therapy. Flow cytometry analysis revealed a high expression of classical mesenchymal markers (CD105, CD90, CD73) and a negligible expression of hematopoietic and immunological markers (CD34, CD45, HLA-DR), validating the mesenchymal origin of the isolated cells. Furthermore, the tri-lineage differentiation potential, into adipocytes, chondrocytes, and osteoblasts, confirmed the multipotency of hDP-MSCs, a key attribute for their application in regenerative therapies.

The therapeutic potential of the hDP-MSC-derived secretome was evaluated in vitro using a scratch assay with HaCaT keratinocytes under high- and low-glucose conditions. We initially evaluated the effect of the secretome in an environment mimicking the conditions of patients with diabetic foot ulcers and then in normal conditions. In both conditions, secretome treatment significantly accelerated wound closure at 24 and 48 h, highlighting its ability to enhance keratinocyte migration, an essential component of re-epithelialization.

The enhanced wound closure observed in the SeCR group compared to the CTR group likely reflects the biological activity of soluble factors secreted by hDP-MSCs. Although the specific components responsible for this effect were not identified in the present study, the current literature supports the notion that these secreted elements orchestrate a coordinated response that promotes re-epithelialization and tissue repair [29,30,31]. This includes the stimulation of keratinocyte migration and proliferation, modulation of local inflammation, and enhancement of matrix remodeling, all key events in the wound healing process [32,33,34]. Thus, the results observed reinforce the finding that the regenerative effect of the secretome extends beyond baseline cell behavior, contributing actively to the acceleration of wound closure. Further investigations aimed at characterizing the secretome’s molecular profile will be essential to validate these mechanisms and optimize its therapeutic potential.

In addition, studies have shown that, in the presence of high levels of glucose (20 mmol/L), the glucose transport rate of proliferating or differentiating primary keratinocytes is downregulated, impairing the energy metabolism of the cells and leading to changes in cell morphology, as well as decreased proliferation [35]. Another study in which human keratinocytes were cultured in 6 mM and 12 mM glucose found that hyperglycemia decreased the proliferation rate and inhibited DNA synthesis and total protein production [36]. Similar phenomena were observed in HaCaT cells cultured in 30 mM glucose and in keratinocytes harvested from the wound margins of diabetic rodents [37,38]. Therefore, these results suggest that the secretome is capable of reversing the pathological conditions caused by hyperglycemia and, under normal conditions, improving keratinocyte proliferation as well. Additionally, the secretome demonstrated no cytotoxic effects and did not impair keratinocyte viability, further supporting its potential as a safe and effective therapeutic agent.

To elucidate the underlying mechanisms, we investigated the expression of key inflammatory and regenerative genes following treatment with the hDP-MSC secretome. Under LPS-induced inflammatory conditions, secretome co-treatment modulated gene expression dynamically. At 6 h, treatment with the secretome led to a reduction in TNF-α expression while concurrently increasing the expression of IL-1β and TGF-β1. This early reduction in TNF-α suggests an anti-inflammatory effect, aligning with prior studies demonstrating MSC-derived secretome’s ability to suppress pro-inflammatory cytokines [30,31].

The elevation of IL-1β in the LPS + secretome group, despite its pro-inflammatory classification, may reflect its dual role in early wound healing, including the promotion of keratinocyte proliferation and recruitment of immune cells. The induction of TGF-β1 under inflammatory challenge suggests the secretome’s capacity to activate key regenerative pathways involved in tissue repair. At 24 h, however, TNF-α and IL-1β levels were further elevated in the LPS + secretome group compared to the group treated with LPS alone, suggesting a possible shift toward a pro-inflammatory profile or the activation of feedback regulatory mechanisms following prolonged exposure. One hypothesis is that the secretome exerts its anti-inflammatory effects acutely, with greater activity during the initial hours after exposure (possibly around 6 h), and that its effect may not persist until 24 h. Consequently, the inflammatory response driven by LPS may prevail, reducing or nullifying the secretome’s initial regulatory effects. Despite this, the continued upregulation of TGF-β1 highlights the secretome’s persistent activation of regenerative signaling.

Furthermore, these cytokines often have contradictory functions, exhibiting both pro-inflammatory and anti-inflammatory roles, with their final effect depending on the context in which they are expressed, the duration of their activity, and interactions with other molecules [39]. IL-1β is essential for the initial inflammatory response that initiates the healing process; however, excessive or prolonged activity of IL-1β can hinder healing by contributing to chronic inflammation and impairing tissue repair. Additionally, IL-1β promotes the proliferation of fibroblasts and keratinocytes, which are essential for collagen synthesis and skin regeneration, respectively [39]. Meanwhile, TNF-α is rapidly released at the wound site, initiating the inflammatory phase of wound healing. This inflammation is crucial for clearing debris, combating infections, and attracting immune cells to the area [6]. However, TNF-α can also stimulate the proliferation of fibroblasts and keratinocytes, which are essential for tissue regeneration. It also promotes the expression of growth factors and antimicrobial defenses [6,40].

VEGFα expression remained unaltered in both time points and conditions, indicating that angiogenic responses may be less sensitive to this secretome preparation or may require different temporal or environmental cues for activation.

Collectively, these results suggest that the hDP-MSC-derived secretome exerts a time- and context-dependent regulatory effect on keratinocytes, promoting wound closure through enhanced migration and regulation of key cytokines involved in inflammation and regeneration.

Importantly, its ability to operate under both normoglycemic and hyperglycemic conditions underscores its potential for application in diabetic wound management. However, the dual modulatory effects observed—particularly with respect to TNF-α and IL-1β—emphasize the need for further investigation into the temporal dynamics and dose-responsiveness of secretome components.

Other studies have shown that the secretome of adipose-derived stem cells accelerates wound closure, stimulates angiogenesis, and modulates inflammation, thereby promoting tissue regeneration in chronic wounds [34]. This effect is mediated by factors such as VEGF, TGF-β1 FGF, PDGF, IGFBP-1/-2, and MCSF, which play roles in cell proliferation, blood vessel formation, and regulation of the inflammatory process [34,41,42]. These findings are consistent with the results of the present study, and support secretome as a promising and accessible option for clinical wound healing applications.

A recent study investigated the therapeutic potential of mesenchymal stem cells (MSCs) and their secretome in an imiquimod-induced psoriasis-like skin inflammation model, demonstrating beneficial effects on lesion resolution. The infusion of MSCs, both unchallenged and previously challenged with inflammatory cytokines, accelerated the healing of psoriatic lesions, reduced epidermal thickness and CD3^+^ T cell infiltration, and modulated the local immune response. These effects were accompanied by the induction of TGF-β1 and the secretion of IL-6 and IL-17A, which are associated with immune regulation and cellular differentiation. The study reinforces the ability of MSC-derived secretome to promote skin regeneration through the modulation of inflammatory and regenerative pathways, supporting the findings of the present work by demonstrating the immunomodulatory and wound healing potential of the secretome in different inflammatory contexts [43].

Future studies should focus on characterizing the specific bioactive molecules within the secretome responsible for these effects, including profiling exosomes and microRNAs, and assessing in vivo efficacy using animal models of acute and chronic wounds. Moreover, understanding the interplay between the inflammatory and regenerative signaling pathways activated by the secretome will be essential for optimizing therapeutic strategies.

Despite the promising results, this study has some limitations. The experiments were performed in a simplified in vitro model that lacks the cellular complexity of cutaneous tissue, excluding interactions with fibroblasts, endothelial cells, immune cells, inflammatory mediators, and extracellular matrix components, all of which are essential for proper wound healing. Moreover, the specific bioactive molecules within the secretome responsible for the effects were not identified, limiting insight into the underlying mechanisms of action. Another important limitation is the lack of data on the duration of secretome activity and its stability in the culture medium; it remains unclear whether treatment should be reapplied every 12 or 24 h to maintain efficacy. These aspects are critical for the development of robust therapeutic strategies, and should be addressed in future studies using more comprehensive, physiologically relevant models.

## 5. Conclusions

These preliminary findings reinforce the functional identity of the human dental pulp mesenchymal stem cell (hDP-MSC) secretome and highlight its potential as a promising cell-free therapeutic agent in tissue regeneration. This study demonstrates that the hDP-MSC-derived secretome significantly enhances keratinocyte migration and accelerates in vitro wound closure under both normoglycemic and hyperglycemic conditions, key features relevant to chronic and diabetic wound healing. Importantly, cytocompatibility assays confirmed that the secretome is non-cytotoxic to human keratinocytes, as it did not impair cell viability at 24 or 48 h of treatment in either glucose condition. These findings support its safety for potential therapeutic use.

Moreover, the secretome modulates the expression of critical inflammatory and regenerative cytokines, including IL-1β, TNF-α, TGF-β1, and VEGFα, in a time- and context-dependent manner, suggesting a dynamic regulatory role in the wound microenvironment. Collectively, these results support the secretome’s therapeutic potential in cutaneous repair.

Future directions of this research include the in-depth characterization of the bioactive components of the secretome, such as exosomes, proteins, and microRNAs, responsible for the observed effects. Additionally, in vivo studies using animal models of acute and chronic wounds are necessary to validate these findings and assess safety, efficacy, optimal dosing, and delivery methods. Understanding the temporal dynamics of the secretome’s action and its interaction with different cell types and tissue components will be crucial for its translation into clinical applications.

## Data Availability

The raw data supporting the conclusions of this article will be made available by the authors on request.

## Supplementary Materials

The following supporting information can be downloaded at: https://www.mdpi.com/article/10.3390/pharmaceutics17080961/s1, Supplementary Figure S1. Effect of the secretome on wound closure in HaCaT cells cultured in low-glucose medium at 0 h, 24 h, and 48 h. (A) Representative images of the scratch assay showing cell migration in the control group (CTR) and the secretome-treated group (SECR) at different time points. (B) Graph depicting the percentage of wound closure after 24 h and 48 h. (C) The effect of the secretome on keratinocyte viability was assessed after 24 and 48 h of treatment using the MTT assay. HaCaT cells were cultured under low-glucose conditions during the entire experiment. Secretome-treated cells exhibited significantly greater wound closure compared to the control group. *n* = 5. ** *p* < 0.01; **** *p* < 0.0001.
