# Supplementary material for: The Secretome of Human Deciduous Tooth-Derived Mesenchymal Stem Cells Enhances In Vitro Wound Healing and Modulates Inflammation
Source: Pharmaceutics. 2025 Jul 25;17(8):961. doi: 10.3390/pharmaceutics17080961 (PMC12388939; doi:10.3390/pharmaceutics17080961)
Supplement: Supplementary file 1 [file pharmaceutics-17-00961-s001.zip › pharmaceutics-3725468-supplementary.pdf]

Supplementary Figure S1

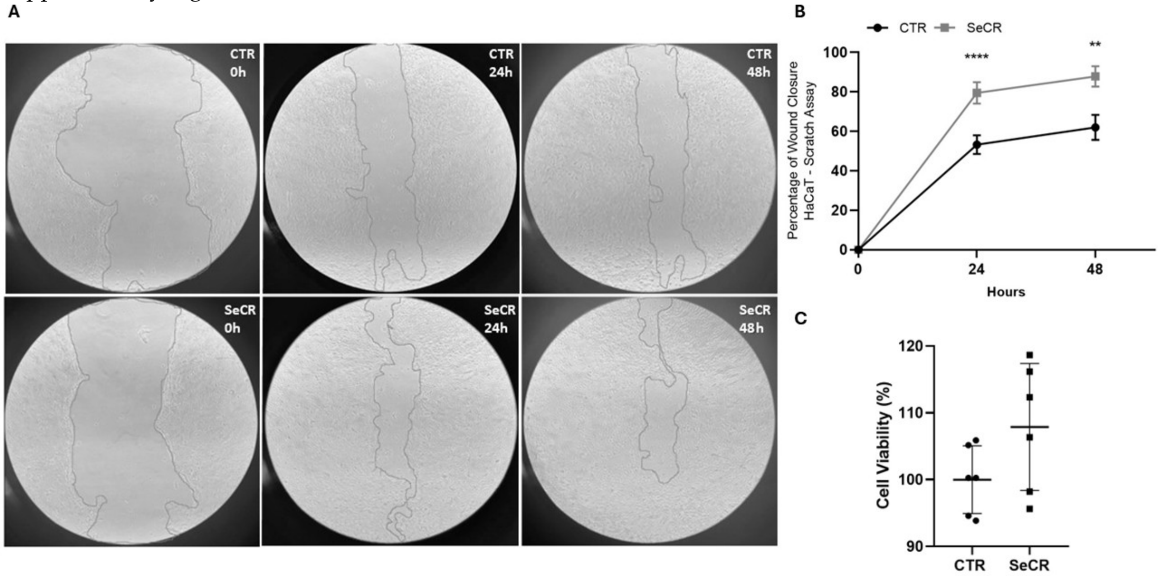

Supplementary Figure S1. Effect of the secretome on wound closure in HaCaT cells cultured in low-glucose medium at 0 h, 24 h, and 48 h. A) Representative images of the scratch assay showing cell migration in the control group (CTR) and the secretome-treated group (SECR) at different time points. B) Graph depicting the percentage of wound closure after 24 h and 48 h. C) The effect of the secretome on keratinocyte viability was assessed after 24 and 48 hours of treatment using the MTT assay. HaCaT cells were cultured under low-glucose conditions during the entire experiment. Secretome-treated cells exhibited significantly greater wound closure compared to the control group. $n = 5$. $^{**}p < 0.01$; $^{****}p < 0.0001$.
